# Atmospheric Pressure Microwave Plasma Jet for Organic Thin Film Deposition

**DOI:** 10.3390/polym12020354

**Published:** 2020-02-06

**Authors:** Mehrnoush Narimisa, František Krčma, Yuliia Onyshchenko, Zdenka Kozáková, Rino Morent, Nathalie De Geyter

**Affiliations:** 1 Research Unit Plasma Technology, Department of Applied Physics, Faculty of Engineering and Architecture, Ghent University, Sint-Pietersnieuwstraat 41, B4, 9000 Ghent, Belgium; Yuliia.Onyshchenko@UGent.be (Y.O.); Rino.Morent@UGent.be (R.M.); Nathalie.DeGeyter@UGent.be (N.D.G.); 2 Faculty of Chemistry, Brno University of Technology, Purkyňova 118, 612 00 Brno, Czech Republic; krcma@fch.vut.cz (F.K.); kozakova@fch.vut.cz (Z.K.)

**Keywords:** atmospheric pressure plasma jet (APPJ), microwave (MW) discharge, thin film deposition, optical emission spectroscopy (OES), Comsol MultiPhysics, methyl methacrylate (MMA), styrene

## Abstract

In this work, the potential of a microwave (MW)-induced atmospheric pressure plasma jet (APPJ) in film deposition of styrene and methyl methacrylate (MMA) precursors is investigated. Plasma properties during the deposition and resultant coating characteristics are studied. Optical emission spectroscopy (OES) results indicate a higher degree of monomer dissociation in the APPJ with increasing power and a carrier gas flow rate of up to 250 standard cubic centimeters per minute (sccm). Computational fluid dynamic (CFD) simulations demonstrate non-uniform monomer distribution near the substrate and the dependency of the deposition area on the monomer-containing gas flow rate. A non-homogeneous surface morphology and topography of the deposited coatings is also observed using atomic force microscopy (AFM) and SEM. Coating chemical analysis and wettability are studied by XPS and water contact angle (WCA), respectively. A lower monomer flow rate was found to result in a higher C–O/C–C ratio and a higher wettability of the deposited coatings.

## 1. Introduction

Atmospheric pressure non-thermal plasma deposition as an advantageous coating technique has been a continually growing research field for over a few decades [1,2,3]. This method has already been frequently used to deposit a wide range of convenient thin films that were previously fabricated by various other methods such as chemical synthesis, electrochemical polymerization, and low pressure plasma-enhanced chemical vapor deposition (PECVD) [4,5,6]. When using the latter methods, controlling the deposition rate, optimizing the structure and adhesion of the produced polymers, reducing the energy consumption, and the enormous cost of vacuum techniques were remaining concerns. Consequently, the field of thin film deposition remains open to alternative methods that can overcome these issues such as atmospheric pressure plasma deposition. Among atmospheric pressure plasma sources suitable for coating deposition, atmospheric pressure plasma jets (APPJs) have gained recognition due to their straightforward design and operation together with their ability to effectively coat 3D complex substrates and their capacity to produce a localized deposition [7,8,9,10,11]. Furthermore, due to its construction, a plasma jet offers the possibility of transporting large amounts of charged and active species while the sample is in indirect exposure to the active discharge, which in turn is helpful for biomedical purposes as well as for the treatment of other sensitive materials [12,13,14,15]. The concentration of reactive species in the plasma directly correlates with the discharge excitation method. The majority of studied atmospheric pressure plasma jets are driven by audio- (typically around 10 kHz) or radiofrequency (usually 13.54 or 27.2 MHz) power supplies and contain a large amount of reactive species [16,17,18,19]. Discharges, generated with even higher excitation frequency (e.g., microwave (MW)), contain an even higher concentration of these reactive species [20,21]. In the case of an MW discharge, the plasma can be (as in the present case) created using the surface wave that propagates along the plasma–capillary or plasma–open-air boundary, and, thus, the active discharge propagates out of the capillary and can also interact directly with the surface. Due to this fact, in an MW-induced discharge, the radicals are present at a high density, thereby contributing to a raised level of fragmentation of the introduced monomer while maintaining room temperature. The plasma torch length can be varied by gas flow and supplied energy [22] and the monomer can be mixed with plasma at different positions with respect to the end of an active discharge. The high radical density in an MW-induced discharge is very advantageous as a high rate of chemical dissociation accompanied by subsequent recombination processes is a crucial reaction for thin film deposition. Furthermore, such a relatively high frequency can also minimize the plasma instability and its transition to arc mode during film deposition, which can bypass the degradation of molecules and chemical bonds during the deposition stage. Nevertheless, despite all the above-listed advantages of MW discharge sources, only a few works have been conducted which have focused on the performance of MW plasma jets in thin film deposition under different operational conditions [8,23,24]. Additional advantages of MW plasma application for thin film deposition are its simplicity and its generally cheap scalability up to a couple of meters. Thus, laboratory achievements could be generally simply extended to industrial scale practice.

A thorough investigation of a complete experiment is required to give a full insight into the processes involved in plasma jet polymerization and properties of the deposited films. Plasma diagnostics in this work are used as a powerful tool to establish the connection between monomer fragmentation/distribution and coating properties, as it is crucial to demonstrate correlations between the plasma discharge and the resultant coatings. This information is profoundly beneficial for future plasma source development and for tuning the experimental conditions in order to obtain plasma-polymerized coatings with specific requirements suitable for a particular application.

Many different monomers can be used for organic thin film deposition by an MW plasma jet. However, each particular monomer has a specific chemical structure, and, thus, requires a particular energy of the plasma reactive species to be successfully deposited as a coating on a substrate. Among the available monomers, methyl methacrylate (MMA) and styrene are very interesting from a technological point of view. A wide range of applications such as humidity sensors, optical biosensors, protective coatings, biomedical applications, and anti-reflection optical coatings require MMA and/or styrene-based coatings because of their ideal plasticity, transparency, electric insulation, and cushioning properties [25,26,27,28]. Taking this into account, this work focuses on the optimization of styrene and MMA coating deposition by an APPJ operating at an MW frequency of 2.45 GHz. This plasma source has been recently developed and has never been applied for thin film deposition before [20,29]. Consequently, this work is a unique example of using an MW source to polymerize styrene and MMA. Initially, the reactive species in the plasma source are characterized using optical emission spectroscopy (OES). In the next step, gas flow dynamic simulations with Comsol MultiPhysics 5.3 are performed to demonstrate the distribution of the monomer during the deposition process [30,31,32]. In doing so, the gas mixing and propagation in the working volume can be prognosticated, which can in turn help to understand the processes that are taking place during plasma deposition. Moreover, the results of the gas dynamic simulations extensively help to interpret other experimentally obtained data such as temperature and incorporation of other species, which can significantly influence the final coating properties [33,34]. In the final part of the study, the physical and chemical properties of the deposited films are also characterized by water contact angle (WCA) analysis and XPS, while the surface morphology and topography are analyzed using SEM and atomic force microscopy (AFM), respectively. By following this research strategy, crucial information regarding atmospheric pressure MW plasma jet effectiveness for thin film deposition will be revealed.

## 2. Materials and Methods

### 2.1. Materials

Plasma deposition experiments were carried out using styrene (99.5%, Brenntag CZ) and MMA (99.9%, Pliska, Brno, Czech Republic) as precursors. Cover slip glasses 25 mm in diameter were selected as substrates. Furthermore, to have a better visualization of the coating lateral dimensions, silicon wafers (Siegert Wafers, Aachen, Germany) were also used as substrates. Argon of 99.996% purity purchased from Linde was used as the main and carrier gas in the discharge.

### 2.2. Experimental Plasma Set-Up

**Figure 1:**
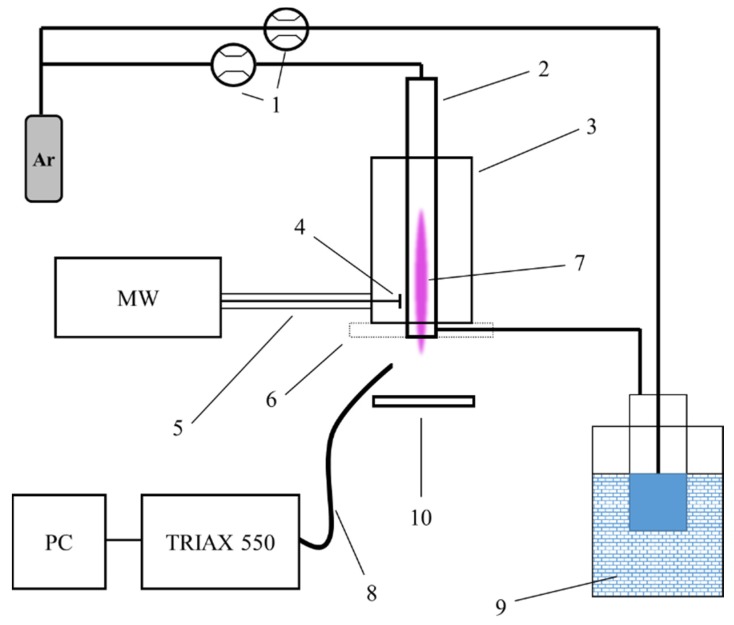
Scheme of the experimental plasma set-up: 1—mass flow controllers; 2—quartz capillary; 3—surfatron resonator; 4—microwave (MW) antenna; 5—MW coaxial cable; 6—Macor plate with precursor injection; 7—plasma torch; 8—optical fiber; 9—bubbler system in water–ice bath; 10—substrate.

A schematic representation of the experimental plasma set-up is depicted in Figure 1. Similarly to a previous study [29], a high voltage power source (Sairem, GMS 200 W) operating with low power at a 2.45 GHz frequency was used for plasma generation. This power supply was connected to a surfatron surface wave (SW) launcher (Sairem, SURFATRON 80, Décines-Charpieu, France) using a coaxial cable. A continuous flow of water was utilized to cool down the surfatron resonator to avoid the influence of elevated temperature. Plasma was generated in a flow of 1 standard liter per minute (slm) argon gas using an Omega FMA-A2408 mass flow controller (Omega, Norwalk, CT, USA) inside a quartz capillary with a 3.06 and 8.0 mm inner and outer diameter, respectively. To introduce the monomer into the plasma, a Macor disc (60 mm diameter and 6 mm thickness) with a cylindrical opening 2 mm in diameter at one side for the monomer carrier gas inlet was placed at the end of the capillary edge. In the middle of this disc there was also a circular opening 4 mm in diameter which ensured that the plasma effluent could freely pass through this opening. Due to the geometry of the MW plasma jet set-up, the precursor was inserted in the plasma afterglow region perpendicular to the main gas stream and towards its core. The Macor disc was also able to improve the efficiency of deposition due to the fact that it alters the gas dynamics in the volume close to the substrate, as previously reported by Onyshchenko et al. [35]. To deliver different amounts of evaporated monomer molecules into the plasma plume, a Bronkhorst F201 mass flow controller was used to regulate an additional argon gas flow, as a monomer carrier gas, with different flow rates (125, 250, 500, and 750 standard cubic centimeters per minute (sccm)) through a glass bubbler filled with MMA or styrene. The bubbler was kept in a water–ice mixture to keep the temperature of the liquid monomers constant. During the plasma deposition experiments, the distance between the bottom edge of the disc and the sample was varied between 3, 5, and 7 mm. For these three distances, the active discharge part of the plasma effluent did not touch the substrate. The other plasma operational parameters that were varied in this work were applied power and deposition time; these were 7, 10, and 13 W and 1, 3, and 5 min, respectively. This work aimed to demonstrate the influence of each of these working parameters on plasma jet performance in terms of thin film deposition. For sake of clarity, one set of experimental parameters (“Main”) was selected as a reference point for all other parameters that were varied. The 10 sets of plasma operational parameters used in this study are summarized in Table 1 and their corresponding labels are also indicated. All deposition experiments were performed in stationary mode, producing only a single plasma treated spot on the substrate to properly verify the deposited area as well as its homogeneity.

### 2.3. OES

OES was used for plasma diagnostics in an effort to reveal more information on the radiative plasma species present in the MW plasma jet. The light emitted by the discharge was integrally collected by a quartz optical cable mounted 5 cm away from the plasma output under an angle of 30° with respect to the plasma jet axis as shown in Figure 1. No additional optical components were used in this case, so no space-resolved OES spectra were collected. For the OES spectra acquisition, a Jobin Yvon TRIAX 550 spectrometer (Longjumeau, France) with an LN2 cooled back-illuminated CCD (1025 × 256, pixel size 26 × 26 µm^2^) was used. Using the standard Ocean Optics DT-MINI-2-GS source (Largo, FL, USA), the system was calibrated with respect to its spectral response. These calibration results were subsequently used for measured spectra correction. The OES spectra were recorded in the range 300–850 nm using a 1200 gr/mm grating at a fixed spectrometer entrance slit of 30 µm. Four integration times (0.002, 0.01, 0.1, and 1 s) were applied for an appropriate signal/noise ratio for each studied line and band.

### 2.4. Computational Fluid Dynamic Simulations

**Figure 2:**
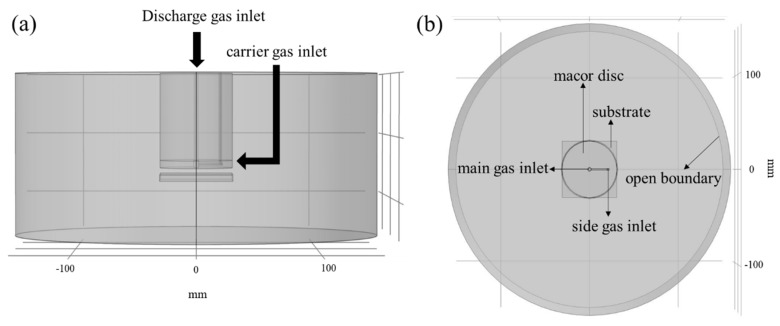
(**a**) XZ-plane and (**b**) XY-plane views of the MW plasma jet geometry.

To obtain information on the impact of the MW plasma jet configuration on the gas dynamics in the plasma region, computational fluid dynamic (CFD) modeling using Comsol MultiPhysics 5.3 was carried out [36]. Considering the design of the experimental set-up and the interaction of the gases with the substrate, simulations were performed under the assumption of turbulent flow [35]. Transport of concentrated species was also applied to obtain information on the distribution of the mass fraction of argon and the precursor. Capillary edge–substrate distances and carrier gas flow rates were selected as variables for computation of the mole fraction and the velocity field in the observation region. Three-dimensional geometry was used for these simulations since there was no axial symmetry in the experimental plasma set-up. Figure 2 represents the constructed geometry of the plasma set-up for the XZ- and XY-plane views. The simulations were carried out in a cylindrical surrounding region with a 150 mm radius and 130 mm height, which was assumed to be filled with ambient air, and the dimensions of all the plasma set-up parts were in accordance with the experimental set-up description of Section 2.2. Argon and a monomer-containing Ar carrier gas were injected from the main gas and side gas inlets, respectively, as shown in Figure 2b, and incompressible flows were considered. For the main gas inlet, a flow rate corresponding to 1 slm was set. For the sideway inlet, the gas flow rate was varied between 125, 250, 500, and 750 sccm. The Navier–Stokes equation and the continuity equation were solved with a fine physics controlled mesh in the volume of interest, i.e.,

(1) $$\rho \left(u.\nabla \right)u=\;\nabla \;.\;\left[-pI+(\mu +\;\mu_{T}\left(\nabla u+{\left(\nabla u\right)}^{T}\right)\right]+F$$

(2) ρ∇ . (u)=0

where ρ is the density (kg/m^3^), u the gas velocity (m/s), I the identity matrix, p the gas pressure, μ the dynamic viscosity (Pa·s), $\mu_{T}$ the turbulent dynamic viscosity (Pa·s), F the volume force (N/m^3^), and ∇ the differential operator.

As previously mentioned, transport of the concentrated species was also introduced, making use of the following equations, i.e.,

(3) $$\nabla .j_{i}+\rho \left(u.\nabla \right)\omega_{i}=R_{i}$$

(4) $$N_{i}=j_{i}+\rho u\omega_{i}$$

where $j_{i}$ kg(m^2^·s) is the relative mass flux component and $\omega_{i}$ a mass fraction.

### 2.5. AFM

The surface topography and the thickness of the deposited coatings were obtained by means of an XE-70 AFM apparatus (Park Scientific Instruments, Suwon, South Korea). In this work, the non-contact tapping mode using a highly-doped single crystal silicon cantilever with a spring constant of approximately 40 N/m was used to collect AFM images. Subsequently, using XEP software, the roughness of the surfaces was calculated. Additionally, to measure the thickness of the deposited films, a scratch was first made with a sharp blade on the surface of the cover slip glass covered by the coating. Subsequently, the scratch region was visualized using AFM and the difference in height formed on the substrate by the scratch was determined using XEP software. Each reported result is an average value calculated from three different measurements and presented with standard deviation.

### 2.6. SEM

After gold sputtering of the coated substrates with a JEOL JFC-1300 Auto Fine Coater (Peabody, MA, USA) for 20 s, the surface morphology of the coatings was visualized using a JEOL JSM-6010 PLUS/LV SEM device (Peabody, MA, USA) which applied an accelerating voltage of 7 kV. The magnifications used to acquire the SEM images in this work were 1000× and 30,000×.

### 2.7. Static WCA Analysis

The wettability of the deposited layers was measured using a Data Physics OCA 10 WCA instrument (Filderstadt, Germany). Droplets of distilled water 1 µL in volume were first placed on top of the deposited coating, after which an image of the water droplet resting on the surface was captured by a CCD video camera (Filderstadt, Germany). Afterwards, by fitting the profile of the water drop using Laplace–Young curve fitting, the WCA value was obtained. The experiments were repeated three times and the measurements were also reported as an average value with an error of less than 3.0°.

### 2.8. XPS

Chemical analysis of the surface of the deposited films was assessed by performing XPS experiments. The measurements were conducted using a PHI Versaprobe II spectrometer (Kanagawa, Japan) with a monochromatic Al K_α_ X-ray source (hν = 1486.6 eV) operating at 25 W, resulting in a beam size of 100 µm. Survey scans and high-resolution C1s spectra were acquired with 187.85 and 23.5 eV pass energy, respectively. All XPS measurements were conducted in a vacuum of at least 10^−6^ Pa with an angle of 45° between the sample surface and the analyzer. By processing the survey scans using Multipak software (v 9.6) and applying a Shirley background, the elemental composition of the samples under investigation was determined. In addition, the high-resolution C1s peaks were curve-fitted by utilizing Gaussian−Lorentzian peak shapes with a 1.4 eV limitation of the full width at half maximum (FWHM) after a calibration of the energy scale was performed with respect to the hydrocarbon component of the C1s spectrum (285.0 eV).

## 3. Results and Discussion

### 3.1. Discharge Characterization

In the first stage of this study, the composition of the plasma during the coating deposition process was investigated. In an effort to obtain better insight into the quantity and quality of excited plasma species and their influence on the properties of the deposited coatings, OES was performed. The following bands and lines were observed in the obtained OES spectra: bands of OH (A→X); N_2_ first negative and second positive systems; and argon, carbon, hydrogen, and oxygen atomic lines. Additionally, a weak radiation of atomic hydrogen was also noticed in the OES spectra. As mentioned above, all spectra were collected using four integration times (1, 0.1, 0.01, and 0.002 s). Nevertheless, in this work, the intensity of each selected component has been presented using an integration time which was sufficiently long to provide a good intensity of signal but which avoided signal saturation at the same time. The evolutions of OH, N_2_ second positive system, N, Ar, Ar^+^, C, CH, and O as some representative lines were selected to assess information on the discharge excited species in correlation with the experimental conditions. Table 2 provides an overview of these selected components with the wavelengths, corresponding transitions, and integration times used for their acquisition. The influence of power and carrier gas flow rate on the intensity of these lines was investigated, since these two parameters had the most significant impact on the concentration of excited species.

**Figure 3:**
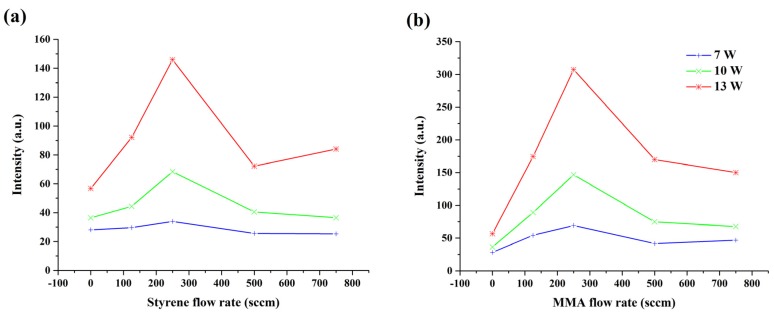
Dependence of the C line emission intensity on the carrier gas flow rate at different powers for the (**a**) styrene and (**b**) methyl methacrylate (MMA) monomers.

The selected spectral lines can be divided into two different groups: one group (OH, N, N_2_ second positive, Ar, Ar^+^, and O) features the discharge itself, and those in the other (C and CH) are representatives of the injected monomers MMA and styrene. The first evident trend observed in all emission spectra was the increase in intensity with increasing MW power, which was due to the extra dissociation and fragmentation of the monomer molecules that subsequently occurred with increasing input power. In fact, higher energy delivered to the plasma resulted in an increased amount of excited species generation. The dependence of the carbon line emission intensity on the carrier gas flow rate at different input powers is presented in Figure 3 for the two monomers under study (styrene and MMA). Based on these graphs, it can be concluded that precursor fragmentation was almost twice as significant when using MMA as a monomer instead of styrene. This difference can be explained by the different levels of dissociation energy required for breaking the bonds in each monomer molecule. Immediately after introducing the monomer to the system, the intensity of the carbon line also increased for both monomers under study. The intensity line sharply increased with increasing carrier gas flow rate until it reached its maximum value at a carrier gas flow rate of 250 sccm. Further increasing the monomer input, however, resulted in a decrease in the intensity of the atomic carbon line, demonstrating that a higher monomer concentration present at the same power level results in a decrease in available fragmentation energy per monomer molecule. Moreover, for a higher carrier gas flow rate, the interaction time between monomer molecules and plasma reactive species was shorter, which in turn decreased the concentration of the carbon species. The same trend with maximum intensity at a 250 sccm carrier gas flow rate was also observed for the CH compound, as can be seen in Supplementary Materials Figure S1.

**Figure 4:**
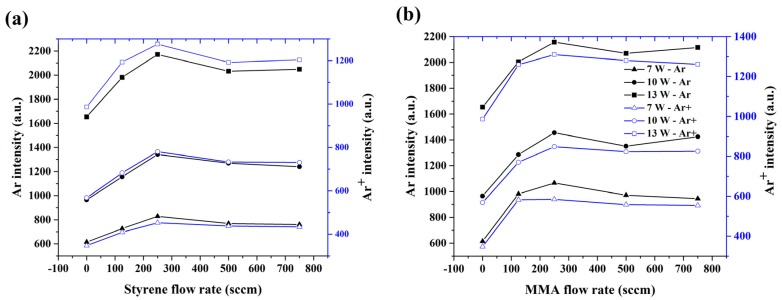
Dependence of the Ar and Ar^+^ line emission intensities on carrier gas flow rate at different applied powers for the (**a**) styrene and (**b**) MMA monomers.

The optical emission of the Ar and Ar^+^ lines is proportional to the concentration of reactive species in the argon discharge. Thus, the dependency of these line intensities on the carrier gas flow rate and applied power can provide insight into the processes occurring during thin film deposition. The evolution of the Ar and Ar^+^ line emission intensity as a function of carrier gas flow rate at different discharges powers is presented in Figure 4a,b for styrene and MMA, respectively. A comparison between Figure 4a,b suggests that for a higher applied power, the intensity of Ar and Ar^+^ were almost independent of the type of monomer which was present in the discharge, while for lower power, an admixture of MMA resulted in an enhanced intensity of Ar and Ar^+^ lines in comparison with styrene. In addition, the signal from these two argon lines increased with increasing carrier gas flow rate and again reached its maximal level at a carrier gas flow rate of 250 sccm for both monomers under study. The growing trend can be explained by the higher amount of overall argon gas in the system which flowed with a higher speed. At a carrier gas flow rate of above 250 sccm, a steady state of the intensity of the Ar and Ar^+^ lines appeared, which can be seen to be related to the air admixing and the presence of a higher concentration of monomer molecules in the discharge. The same trend of intensity profiles was also observed for the N_2_ second positive, N, OH, and O bands (see Figures S2–S5, Supplementary Materials). The similarity in the optical emission intensity between all these mentioned bands and the argon lines thus indicates that the processes standing behind the excitation were strongly interconnected.

Making use of the integral intensity of a few selected argon lines, namely the lines at 603.2, 675.3, 687.1, and 714.7 nm, the excitation temperature of argon was also calculated using corresponding constants from the NIST (National Institute of Standards and Technology) database [37]. The calculations resulted in excitation temperatures in the range 4300–5000 K under all experimental conditions and for both monomers. In general, no dependence on the carrier gas flow rate was observed for the excitation temperature. However, the excitation temperature was slightly higher with increasing power. In the next step, a Boltzmann plot from the N_2_ second positive 0-2, 1-3, and 2-4 band heads was used to determine the vibrational temperature. This temperature was found to be dependent on discharge power and monomer type and was rather stable when changing the Ar carrier gas flow rate. For the MMA precursor, the vibrational temperature varied between 2100 and 2900 K depending on the applied power while this temperature range was slightly lower for styrene, being 2000–2800 K. Furthermore, according to the Boltzmann distribution, using the N_2_ second positive 0-2 band with J = 27–32, the rotational temperatures were also calculated [38]. For this calculated rotational temperature, no significant impact of power, monomer type, and carrier gas flow rate variation on the obtained value was observed. The range of rotational temperature variation for both monomers was found to be 1250–2500 K, with a mean uncertainty of 250 K. Additionally, the rotational temperature calculated from the OH radical lowest lines (details in [20]) was 550–700 K, with a mean uncertainty of 70 K.

### 3.2. CFD Simulations

Additional to the plasma characterization, 3D numerical simulations of fluid dynamics were also carried out in this study to obtain a basic perception of how the mixing of the main Ar and the Ar carrier gas stream occurred. Moreover, information on the distribution of the monomer in the volume close to the substrate was also able assist in evaluating the thin film growth during the deposition process. For example, it has already been shown that the gas velocity and mass fraction can be correlated to the deposition pattern on a substrate [39] and the plasma behavior [35]. In the latter study it was also revealed that placing an additional plate at the edge of the plasma jet capillary intensified the gas purity near the surface when working with a small gap distance to the substrate [35]. In this study, the experimental set-up was quite similar to that used in [35], as the additional disc for the purpose of monomer introduction also played the role of an additional plate and thus constrained the gas in a small volume, which in turn influenced the gas diffusion and propagation near the substrate. All computations in this work were only performed for the styrene monomer admixture, since its deposition pattern on the substrate differed from a simple circular shape. This was not the case when using MMA as monomer, as will be shown in the following sections.

**Figure 5:**
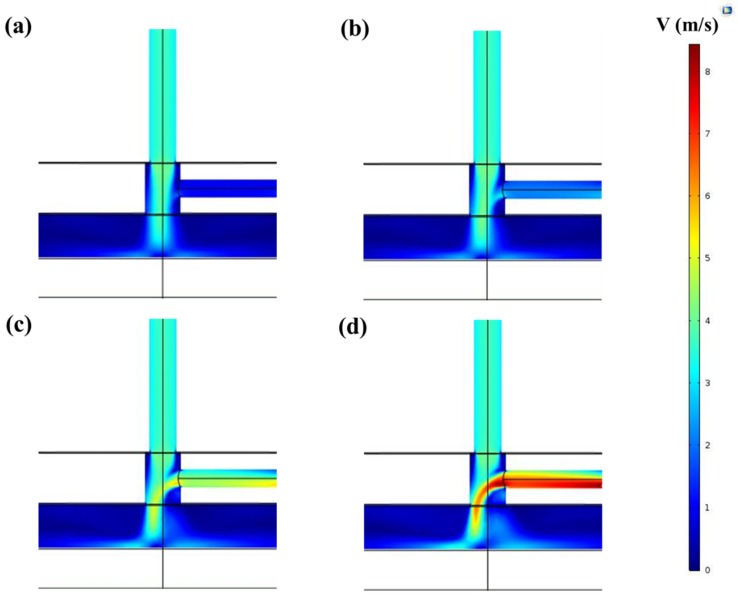
Velocity distribution (m/s) in the XZ-plane at a 5 mm gap size between the capillary and the substrate for a sideway gas flow rate of (**a**) 125 sccm, (**b**) 250 sccm, (**c**) 500 sccm, and (**d**) 750 sccm.

The first stage of the simulations was focused on defining the velocity distribution in the MW plasma jet system. In this step, the main gas flow rate was fixed at 1 slm and the distance between the capillary and the substrate was set to 5 mm. Computations were performed for different monomer-containing gas flow rates (125, 250, 500, and 750 sccm), and Figure 5 demonstrates the gas velocity for these different gas flow rates in the XZ-plane. Figure 5a shows that for the smallest value of the carrier gas flow, there was no disturbance to the main gas stream, as a higher velocity was present in the larger channel compared to the sideway gas inlet. Figure 5b also shows that when the additional gas reached a flow rate of 250 sccm, the velocity of the two gas streams became comparable at their meeting point. Moreover, when using even higher carrier gas flow rates (Figure 5c,d), the impact of the sideway gas inlet became more pronounced. Under these experimental conditions, the side gas flow could not easily deviate from its direction and could not mix well with the main gas at their meeting point. The higher velocity of the sideway gas pushed the monomer-containing gas and the main gas flow on the side of the capillary opposite to the sideway gas inlet, as shown in Figure 5c,d. Changes in the velocity of the gas near the substrate can be seen as well in Figure 5. The homogeneous velocity distribution of the gas in the case of small side gas flow rates changed to an uneven distribution when injecting sideway gas with higher flow rates. It is also worth mentioning that the diameters of the two gas inlets were different (3.1 mm for the main gas stream and 2 mm for the sideway gas inlet), which influenced the velocity magnitude. The high velocity of the main argon gas stream in the center could also prove that there should be a negligible amount of deposition in the center of the substrate, especially when using low side gas flow rates.

**Figure 6:**
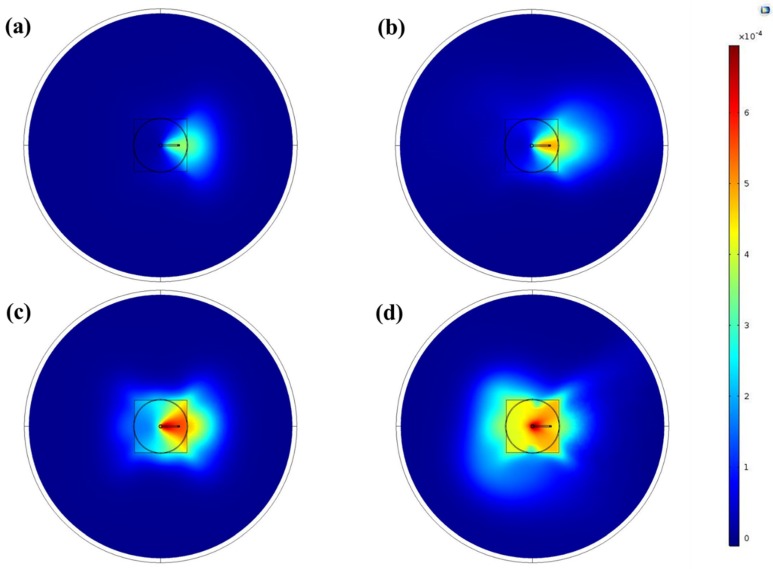
Top view (XY-plane) of the monomer mass fraction distribution at 5 mm gap size for (**a**) 125 sccm, (**b**) 250 sccm, (**c**) 500 sccm, and (**d**) 750 sccm side gas flow rates.

**Figure 7:**
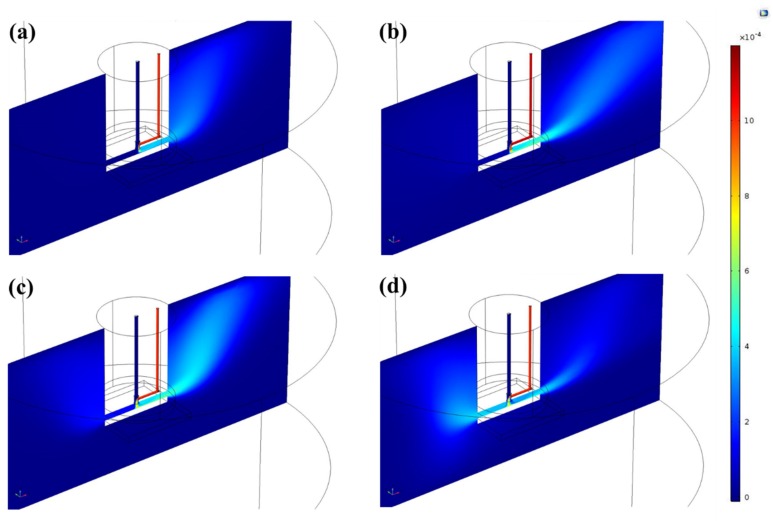
XZ-plane cross-sections of the monomer mass fraction distribution at 5 mm gap size for (**a**) 125 sccm, (**b**) 250 sccm, (**c**) 500 sccm, and (**d**) 750 sccm side gas flow rates.

In a next step, the mass fraction of the monomer precursor and the way the monomer propagated on the surface were simulated. The results are shown in Figure 6 for the XY-plane (top view) and in Figure 7 for the XZ-plane of the used 3D model. The XY-plane crossed the volume at the position where the substrate surface was located during the experiments. The shown series of graphs indicates that for the smallest carrier gas flow rate (125 sccm), only a small amount of monomer was present on the substrate surface and that the monomer completely diverged to the right-hand side of the plasma set-up. An increase in the monomer-containing gas flow rate to 250 sccm intensified the presence of the monomer at the right side of the set-up with only the presence of a negligible amount of monomer on the other side of the substrate. On the other hand, when the flow rate of the side gas was further increased to 500 sccm, a small propagation of the monomer to the left side of the substrate was observed. Moreover, a 750 sccm side gas flow rate even led to the monomer spreading over almost the complete area of the substrate. In the XZ-plane of the 3D model, depicted in Figure 7, the majority of the monomer also deviated to the right-hand side below the opening of the Macor disc on the substrate surface when the carrier gas flow rate was small (Figure 7a,b). For these studied conditions, the velocity of the main gas stream was much higher than the velocity of the gas stream coming from the smaller side channel. Consequently, the monomer only slightly mixed with the main stream of the system and remained on the same side when the gas outflowed the capillary. On the other hand, in the cases where carrier gas flow rates of 500 and 750 sccm were used (Figure 7c,d), the mixing process of the two streams was significantly improved and the monomer was more directed to the center, since it was entering the channel with a higher velocity. Moreover, at the highest flow of the side gas, the volume between the Macor disc and the substrate was almost entirely filled with monomer, which was not the case for the other analyzed carrier gas flow rates. It can therefore be concluded that increasing the carrier gas flow rate resulted in a more uniform monomer dispersion on the substrate surface based on the mass fraction of the precursor in the volume between the plasma jet and the substrate. These results of the monomer propagation region shape can thus provide a preliminary prediction of the footprint of the deposited layer on the substrate.

**Figure 8:**
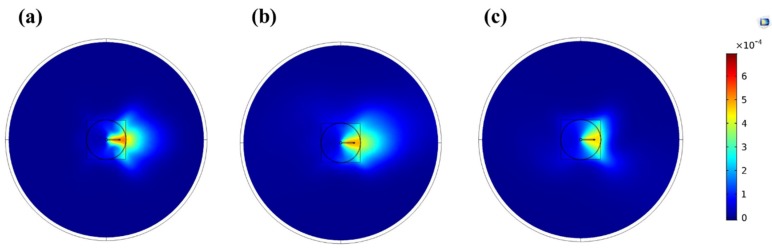
Top view (XY-plane) of the monomer mass fraction distribution for (**a**) 3 mm, (**b**) 5 mm, and (**c**) 7 mm gap sizes. The monomer-containing gas flow rate was fixed at 250 sccm.

The size of the volume between the Macor disc and the sample was regulated by the gap size between these two planes. Consequently, the working distance also had a significant influence on gas propagation at the capillary outlet. Figure 8 shows the influence of the gap size on the monomer distribution for distances of 3, 5, and 7 mm between the bottom of the Macor disc and the substrate plane. In these simulations, the sideway gas flow rate was fixed at 250 sccm. For a smaller distance (Figure 8a), the volume of propagation was too small to provide a sufficient distance for efficient mixing of the two gas streams. As a result, the pattern of the monomer fraction was in this case mainly controlled by the main gas flow. A similar behavior was also observed when using a 5 mm distance, yet a more uniform gas propagation on the right side of the sample was present due to a longer available distance for mixing of the two gas streams. Finally, for a 7 mm distance between the disc and the substrate (Figure 8c), the mass fraction of the monomer was more evenly distributed on the surface of the substrate, which might in turn have resulted in a better homogeneity of the deposited coating.

### 3.3. Physical and Chemical Surface Characterization

#### 3.3.1. Coating Appearance and Homogeneity

**Figure 9:**
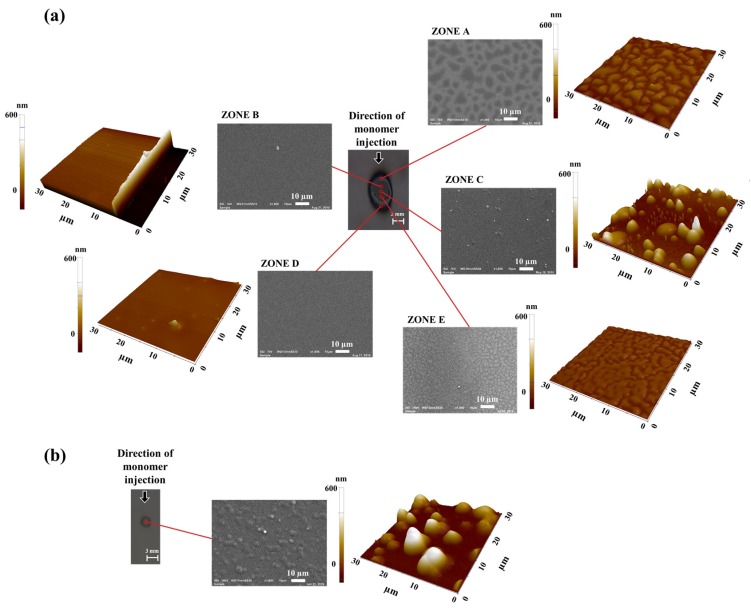
Overview of the appearance, surface morphology, and surface topography of thin films deposited with the MW plasma jet by injection of the (**a**) styrene and (**b**) MMA monomers. The observed area of the 3D atomic force microscopy (AFM) image is 30 × 30 µm^2^.

In the next step of this study the physical and chemical properties of the deposited coatings were investigated. For this purpose, the MW plasma jet was used for thin film deposition of MMA and styrene coatings. Initially, both monomers were deposited on a silicon wafer, making use of the main conditions (power: 10 W, monomer-containing gas flow rate: 250 sccm, deposition time: 3 min, and gap size: 5 mm (see Table 1)) to get an idea of the deposited thin film pattern on a flat substrate. The obtained plasma-deposited thin films were studied in terms of surface morphology and topography by utilizing SEM and AFM, respectively, and the obtained results have been depicted in Figure 9. As can be seen in this figure, each coated silicon wafer had a unique appearance depending on the used monomer and the way it was mixed into the MW plasma jet effluent. The diameter of the coated area for the plasma-deposited styrene was approximately 20 ± 5 mm, while for MMA, the diameter was reduced to 3.0 ± 0.5 mm. However, in the case of styrene, the deposition pattern on the substrate was not entirely symmetrical. This result can be explained by the non-homogeneous mixing of the monomer into the main stream of the plasma, as was shown in the previous section. On the other hand, when MMA was added to the plasma, a coating located near the center of the substrate was deposited, as demonstrated in Figure 9b. Figure 9 thus clearly shows that the styrene coating had larger dimensions compared to the MMA-based film deposited under the same experimental conditions. A possible explanation for this dissimilarity may be the difference in deposition rate of the two monomers under study. For instance, organic compounds containing double bonds and aromatic rings are known to have a higher deposition rate in comparison to branched-chain or non-aromatic structures [40]. Hence, styrene is expected to show a higher deposition rate than MMA, which can in turn result in a larger deposition area.

On the styrene-based coating area, five different zones have been determined which all showed a different surface topography and morphology. Based on Figure 9a, the following zones were selected: zone C—directly under the MW plasma jet capillary where the coating appeared in the form of dispersed droplets with round shapes and sizes reaching up to 1 µm; zone B—located above zone C, consisting of a very smooth and thick coating; zone D—located below zone C where no deposition at all could be observed; and zone A and zone E—located at the edges of the coated area. In these last two regions, the deposits were characterized by a very thin non-uniform coating that contained plenty of vacant spots. The location of the MMA coating, as shown in Figure 9b, matched the position of zone B of the styrene-based sample, suggesting similarity in gas dynamics for both monomers as the same plasma jet device was used. Indeed, a higher amount of deposited precursor was situated above the center of the substrate as the monomer gas injection point was located in this zone. This not-centered position of the deposited coating can be explained by the suppressive behavior of the main gas stream towards the injected monomer. Since the monomer is injected in the region where the plasma jet is fully developed, well-structured, and close to the outlet, it does not have enough residence time as it propagates towards the core of the plume. Thus, the majority of the monomer is not deposited in the central area but in the zone shifted towards the monomer injection side. Figure 9b also shows that the coating morphology was different when MMA instead of styrene was used as a monomer, which could be attributed to the lighter weight of MMA molecules compared to styrene. The OES results suggest that the plasma jet with injected MMA contained a higher amount of excited species which could have interacted with the monomer molecules. This could in turn have led to nucleation in the gas phase, which is followed by condensation and finally deposition in the form of droplets on the surface. This effect was also valid for styrene but was less pronounced, as shown in Figure 9a.

#### 3.3.2. Coating Morphology

**Figure 10:**
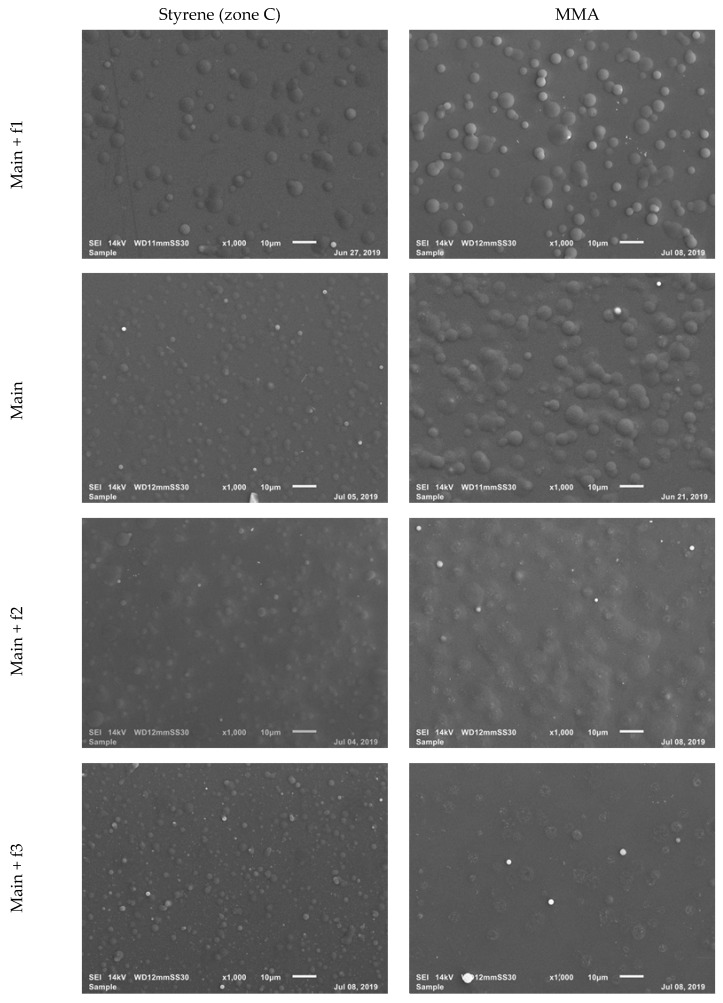
SEM images of the central zone of styrene (zone C) and MMA coatings deposited with different carrier gas flow rates.

In this part of the study, the morphology of the coatings was examined using SEM imaging. As mentioned above, the majority of the coated sample surfaces contained droplets distributed all across of the deposit. Figure S8 (Supplementary Materials) depicts a cross-sectional view of the coatings on the glass substrate under the main conditions. The formation of droplets and clusters on the sample surface is a sign that monomer plasma polymerization actually occurs during the coating deposition [25,41,42]. Based on the used experimental conditions, the size and distribution of these deposited droplets were found to fluctuate. Figure 10 shows the SEM images of both styrene (zone C) and MMA samples prepared with different carrier gas flow rates. Generally, deposited styrene in the center contains droplets with a smaller size compared to the MMA monomer for all experimental conditions. A common trend can also be noticed for the SEM images presented in Figure 10: increasing the monomer-containing gas flow rate caused an increase in the number of droplets formed on the surface of the coatings. Additionally, by adding monomer to the system the size and shape of the droplets also varied. For example, when using 750 sccm MMA, the large amount of injected monomer resulted in a surface coverage by a high amount of droplets which appeared as a smooth coating. The influence of other working parameters (time, power, and distance to substrate) on the surface morphology can be observed in Supplementary Materials Figures S6 and S7 for MMA and styrene, respectively. A short deposition time (1 min) and lower carrier gas flow rate resulted in coatings possessing a smaller amount of droplets, while higher values of these parameters produced deposits with a higher amount of droplets across the investigated area for both monomers. In addition, the distance between the MW plasma jet disc and the substrate influenced the size of the droplets: increasing this distance to 7 mm made the size of the deposited droplets larger. This can be explained by the gas dynamics in the volume between the Macor disc and the substrate. A smaller distance can provoke vortex creation at the center [14], which can affect the location of droplet creation on the substrate surface, while for a larger distance this effect is less prevalent, resulting in deposits with larger-sized droplets. To prove that a plasma-polymerized cross-linked coating was deposited on the substrates in this work, the coatings were submerged in water for a short duration. This was tested for the main condition deposits and Figure S9 (Supplementary Materials) confirms that the coating was still present on the substrate after immersion in water for 24 h. This result clearly evidences that a plasma-polymerized cross-linked coating was deposited in this work [43,44].

#### 3.3.3. Coating Thickness

As previously mentioned, to measure the thickness of the deposited film, a scratch was first made with a sharp blade along the zones of the samples that were defined on the surface of the coating deposited on the cover slip glass. Subsequently, the scratch region was visualized using AFM and the difference in height formed on the substrate by the scratch was determined. Within this context, it is important to mention that during the making of the scratches, the cover slip glasses remained untouched, making the thickness measurement more reliable. As previously shown in Figure 9, it had already been observed that a thick homogeneous coating was obtained in zone B of the plasma-deposited styrene sample. As a result, in the case of styrene, the thickness of the films was measured in zone B. Coating thickness measurements were, however, not performed for samples prepared using MMA as a monomer, as in this case the coating was only spread across the substrate in the form of droplets. Consequently, Table 3 only contains the coating thickness results for styrene samples prepared under different experimental conditions.

These results demonstrate that the lowest measured thickness corresponded to the lowest deposition time (1 min), and, conversely, that the highest deposition time resulted in the thickest coating. Another parameter that had a direct influence on the coating thickness was the carrier gas flow rate. As is reported in Table 3, the smallest Ar flow-carrying styrene (125 sccm) produced a coating with a 110 nm thickness after a deposition time of 3 min. When a higher Ar carrier gas flow rate of 250 sccm was used, the coating thickness reached its maximum level of 229 nm after the same deposition time. Interestingly, any further increase of the carrier gas flow rate did not result in a thicker coating but resulted in a more homogenous film deposition over the substrate area as other measurements along the scratch in this zone showed only a small deviation from the reported coating thickness. Based on the simulation results reported in Section 3.2, it was concluded that at the highest carrier gas flow rate (750 sccm), the carrier gas was injected into the system with a considerably high velocity, which could in turn spread the monomer all over the substrate surface due to efficient mixing with the main plasma jet gas stream. Besides the gas propagation behavior, which only predicted a better uniformity of the coating at the highest carrier gas flow rate, the available energy for monomer fragmentation should also be taken into account to explain why the coating thickness did not increase when increasing the Ar carrier gas flow rate to 500 and 750 sccm. Since the applied power remained the same while the carrier gas flow rate was varied, the average energy per monomer molecule reduced with increasing monomer injection flow. As a result, the efficiency of precursor fragmentation was also less pronounced at higher sideway gas flow rates, which is in agreement with the OES results shown in this work. Although one would expect a thicker coating with increasing carrier gas flow rate as a higher amount of monomer was injected in the system, this increase in monomer amount was in this study counteracted by the lower amount of monomer fragmentation, ultimately leading to the deposition of a thinner coating.

Table 3 also reveals that the distance between the disc and the substrate also influenced the thickness of the coating: a higher distance to the substrate yielded a thicker and more uniform coating, while a smaller gap size provided a wider coated area with a lower coating thickness and less uniformity. The latter conclusion concerning coated area and coating uniformity was drawn based on thickness measurements which were performed along the scratch. These results can again be well correlated with the simulations performed in this work, showing the effect of distance on the monomer distribution. Moreover, at a lower distance, a developed turbulent flow of main and carrier gas can occur, which can in turn influence the size and homogeneity of the coated area. Finally, Table 3 also shows that the coating thickness was also affected by the applied power. An increase in power to 10 W resulted in a higher coating thickness, resulting from more monomer fragmentation at elevated power. However, at a power of 13 W, the coating thickness was not increased further, which could be explained by the fact that in this case monomer fragmentation was too pronounced to efficiently deposit a coating.

#### 3.3.4. Coating Roughness

Another critical coating parameter that can be measured by AFM is surface roughness, and the obtained roughness results are presented in Table 4. Generally, for styrene samples, the highest surface roughness was observed in the center of the deposit (zone C), as in this area the monomer was mostly deposited in the form of dispersed droplets with varying sizes. This roughness, however, decreased in zone B in comparison to central zone C, as indicated by the results shown in Table 4. Noting that the roughness for the pristine glass is 5 nm, zone B did contain a coating, and this coating was quite smooth, as the roughness varied between 14 ± 2 and 32 ± 6 nm, depending on the experimental conditions. These results are thus consistent with the AFM image of zone B, shown in Figure 9a. After this smooth coating region, an area covered by a non-homogeneous coating with different vacant sites was observed to follow (zone A). In this region, the roughness was found to be rather similar to or slightly higher than the roughness values obtained in zone B, depending on the applied experimental conditions. Table 4 also reveals the influence of deposition time on the surface roughness: for both monomers, an increase in deposition time resulted in a higher surface roughness. This finding can be attributed to a higher number of accumulations of the monomer on the surface at random locations when using longer treatment duration. Consequently, this monomer distribution in the form of droplets resulted in an increase in coating surface roughness. Another experimental parameter that was found to influence the surface roughness was the applied power: for both monomers, an increase in power resulted in a rougher deposit. This can be explained as follows: at a higher power, a higher level of monomer fragment reactions occurred in the gas phase instead of on the gas–substrate interface, thereby leading to a rougher coating surface. In contrast to deposition time and power, the influence of carrier gas flow rate on the surface roughness was dependent on the type of injected precursor (MMA or styrene). For MMA, the highest surface roughness was obtained when using the lowest carrier gas flow rate, and this roughness decreased with increasing flow of monomer into the system. As stated above, with an increase in the amount of injected monomer to the system the droplets on the substrate recombined with each other and decreased the surface roughness and finally completely covered the surface at the higher flow rate, producing a smooth coating. In the case of styrene, the smallest carrier gas flow rate of 125 sccm produced films with a comparatively small roughness in all zones. These roughness values in the center of the sample (zone C) increased when a 250 sccm monomer-containing gas flow rate was used, and then again decreased when this gas flow rate was further increased to 500 and 750 sccm. This behavior can be explained by a combination of monomer distribution and gas velocity during the deposition process. Zone C is just below the plasma jet capillary, and, thus, with a 125 sccm carrier gas flow rate, the monomer was not well mixed with the main stream, leaving this area almost without a coating (just a few deposited droplets). A higher amount of droplets was able to be observed for the next value of Ar carrier gas, since in this case more monomer fragments were delivered to the surface by the faster gas stream. Lastly, a smooth layer in zone C was observed when deposition was performed at higher flow rates (500 and 750 sccm) due to good monomer mixing with the main gas stream and its uniform distribution under the plasma jet capillary, as was shown in Section 3.2. In zones A and B, which were located at the side where the monomer was injected, the values of surface roughness increased with increasing monomer-containing gas flow rate. These findings can be explained by the simulation results obtained earlier. It was shown that at a lower flow rate the monomer was not well mixed with the main stream and mostly remained on one side of the plasma jet, while higher values of carrier gas flow rate caused direct injection of the monomer into the core of the MW jet. Thus, the nucleation of styrene near zones A and B proceeded at different radial positions of the plasma jet: near its edge or in the core for smaller and higher side gas flow rates, respectively. This different behavior resulted in coatings with different roughness values at these positions on the substrate, depending on the amount of injected monomer. Finally, the distance between the MW plasma jet and the sample was found to have an insignificant influence on surface roughness.

#### 3.3.5. Coating Wettability

In this part of the study, the wettability of the deposits prepared in this work was determined utilizing WCA analysis. The measurements were again performed on the styrene and MMA samples in the zones that were introduced earlier in this work. The wettability of an uncoated cover slip glass was measured to be 79° ± 2°. After plasma deposition, the WCA values of the coated samples significantly decreased, suggesting that the coated layers were hydrophilic. Summarized results of WCA measurements for both monomers and under different experimental conditions are presented in Table 5. These results indicate that shorter deposition times and smaller carrier gas flow rates resulted in coatings with higher hydrophilicity for both precursors. Consequently, a higher flow rate of the monomer-containing gas produced deposits with higher WCA values. This latter behavior can be explained by a reduction in monomer molecule fragmentation, and, therefore, an incorporation of more non-oxidized long-chain monomers on the surface with increasing carrier gas flow rate. Furthermore, Table 5 also shows that an increase in applied power resulted in more hydrophobic coatings. A possible explanation for this can be that there was lower fragmentation with lower power. In the case of styrene, the highest wettability was for all experimental conditions observed in zone C, the location precisely under the plasma exposure point, which might be due to the fact that this point had the best interactions with the plasma jet during the deposition process. However, while radially proceeding to the edge of the sample, in the direction of zone A, a reduction in coating wettability was observed for all experimental conditions under which styrene coatings were deposited. Only a small difference in WCA was observed for different distances between the MW plasma jet and the substrate, which can be explained by variations in the gas flow dynamics in the volume above the sample.

#### 3.3.6. Coating Chemical Composition

Chemical surface analysis is one of the most valuable characterization tools for deposited coatings, as it provides information on thin film surface composition. The elemental compositions of the coatings prepared under various experimental conditions were determined by XPS in addition to the concentration of the carbon-containing chemical bonds. Similarly to WCA, the coatings were again analyzed in the same zones as where all other measurements were performed. From XPS survey spectra it was found that the coatings produced from styrene only consisted of carbon and oxygen, except for the central point (zone C), at which a small amount of nitrogen was also detected. It is well known that APPJ treatment can induce the incorporation of oxygen by post-treatment exposure of the sample to the atmosphere or by reactions with oxygen which are present in the discharge due to gas impurities and ambient air admixture [45,46]. OES measurements demonstrated that in this work a considerable amount of oxygen and nitrogen species were present in the plasma jet during the thin film deposition when using styrene as a monomer. This can explain why in case of styrene, which contains no oxygen in its chemical structure, it was possible to detect oxygen and nitrogen in the deposited coatings. In the case of MMA, the surface of the thin films only consisted of carbon and oxygen, regardless of the location or the applied experimental conditions, which is in agreement with the chemical structure of the original monomer. Based on the elemental composition results, O/C ratios were calculated for MMA and all regions of the styrene samples, and the obtained values have been presented in Supplementary Materials Table S1. These results were found to be in good agreement with the earlier obtained WCA results, since a higher oxygen incorporation (higher O/C ratio) corresponded to a more hydrophilic region. Moreover, for the styrene samples, the O/C ratio followed the same trend across the surface as was noticed before for the coating wettability properties. A higher amount of oxygen was incorporated in the central area (zone C), and each subsequent area towards the edge of the coating contained less oxygen.

**Figure 11:**
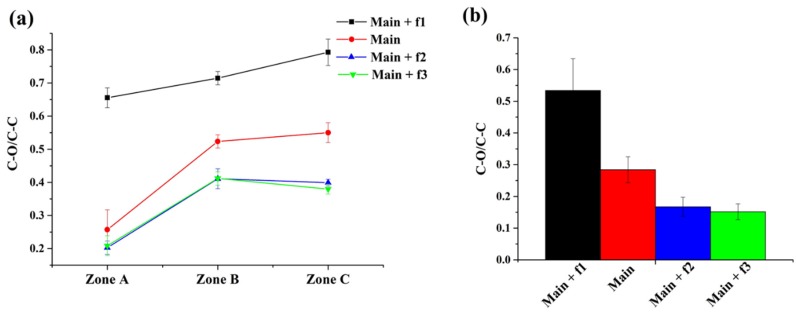
Influence of carrier gas flow rate on C-O/C-C ratio for (**a**) styrene and (**b**) MMA samples.

Deconvolution of high-resolution C1s spectra was also performed to obtain more detailed information on the chemical structure of the deposited thin films. The C1s high-resolution spectra for deposited styrene coatings were fitted with five curves, namely, (1) aromatic C–C/C–H bonds at 284.8 eV; (2) aliphatic C–C/C–H bonds at 285 eV; (3) the oxygen-containing group C–O at a binding energy of 286.6 eV; (4) O–C–O/C=O groups at a binding energy of 288.1 eV; and (5) a shake-up peak at 291.5. For MMA samples, curve-fitting of C–C/C–H, C–COO, C–O, O–C–O/C=O, and O–C=O were used at binding energies of 284.8, 285.4, 286.6, 287.9, and 289.1 eV, respectively. Figure S10 (Supplementary Materials) shows a typical deconvolution of the detailed C1s spectra for styrene and MMA deposits with the indication of the reference peaks ascribed to conventional PS (polystyrene) and PMMA (poly(methyl methacrylate)) as well as new peaks induced during plasma polymerization [47]. The discrimination of the standard peaks attributed to the mentioned polymers is evidence that the plasma polymerization process was happening during the coating deposition with MW APPJ for both monomers. For the MMA coatings, the appearance of the peak at 287.9 eV could have been due to the scission of C–OCH_3_ bonds during plasma exposure, as was reported in the literature [48]. In the case of styrene, the presence of two extra peaks attributed to oxygen-containing groups suggests the breakage of C–C/C–H bonds, followed by recombination with oxygen from the surrounding environment [49]. The presence of the minor shake-up bond revealed that by using the MW plasma jet for thin film deposition, the benzene ring of the styrene monomer was at least partly preserved in the deposits. Figure 11a,b show the C–O/C–C ratio as a function of carrier gas flow rate for styrene and MMA, respectively. From these figures, it is clear that for both monomers the amount of C-O functional groups was highly dependent on the rate of monomer injection into the system. In all regions of the styrene coating and in case of the MMA coating, the smallest flow rate of the carrier gas (125 sccm) resulted in the highest values of C–O/C–C ratio. Increasing the carrier gas flow rate up to 500 sccm led to the incorporation of a higher amount of carbon in the form of C–C/C–H bonds and, consequently, the C–O/C–C ratio decreased. Further increasing the carrier gas flow rate to 750 sccm, on the other hand, did not significantly affect the C–O/C–C ratio anymore for both monomers under study. This trend can be explained by the concentration of the monomer in the discharge during its fragmentation: at a lower carrier gas flow rate, fragmented monomer molecules mostly interacted with oxygen molecules present in the plasma rather than with other monomer fragments which occurred at higher rates of monomer injection. Hence, the C–O/C–C ratio was lower in the case of higher carrier gas flow rate due to more pronounced recombination processes between the monomer fragments. The influence of the applied power, the distance between the plasma jet disc and substrate, and the deposition time on the coating chemical composition was also investigated and the results have been reported in Figure S11 (Supplementary Materials) together with the carrier gas flow rate results for both styrene and MMA. This figure reveals that the deposition time and the jet-substrate distance had a mostly insignificant impact on the C–O/C–C ratio: generally speaking, all these samples only varied in a small range of C–O/C–C ratio with varying time and distance. By contrast, the discharge power was found to have an impact on the C–O/C–C ratio: in the case of both monomers, a higher C–O/C–C ratio was obtained for a lower applied power. This result could be explained by the presence of other oxygen-containing functional groups such as C=O and O-C=O at higher applied powers. Finally, Figure 11 and Figure S11(Supplementary Materials) also reveal that for all applied experimental conditions, zones B and C had the highest C–O/C–C ratios, suggesting that the C–O functionalities were mainly present in the region close to the MW plasma jet.

## 4. Conclusions

In this study, the feasibility of MMA and styrene deposition by means of a MW APPJ has been investigated. For the first time, this type of plasma source has been applied to deposit thin layers under atmospheric pressure. Using different techniques, the effect of process parameters such as applied power, carrier gas flow rate, distance from capillary to the substrate, and treatment time on the deposition efficiency has been studied. Firstly, using OES, a MW plasma jet was characterized in terms of excited species. The results demonstrated that a higher amount of reactive species was present in the plasma containing MMA as the monomer compared to the plasma jet containing styrene. It was also observed that the Ar and C bands had a well-defined maximum of OE spectra intensity which was reached when adding 250 sccm of the monomer-containing gas into the system. It can therefore be concluded that the optimal fragmentation of the introduced monomers and argon atom excitation occurred under mid-experimental conditions. CFD simulations were also employed to determine the pattern of precursor propagation in the volume close to the substrate surface. The obtained results of the CFD simulations displayed a remarkable resemblance between the pattern obtained in the experimental stage and the CFD simulations. It was shown that the injection of the monomer from one side caused non-uniformity of the coating due to the non-homogeneous precursor distribution in the examined system. Additionally, an increase in carrier gas flow rate also influenced the pattern of the deposited layer on the substrate and enforced the monomer to propagate further into the main gas stream, thereby providing better mixing. As a conclusion, CFD simulations thus predicted the distribution of the coating on the substrate during the experiments. Coating thickness, roughness, and topography were evaluated using AFM measurements and SEM imaging. The results demonstrated the appearance of droplets in the central area of the deposited styrene coatings while the areas further away from the center showed almost empty regions in one direction and a comparatively thick coating layer in the other direction due to monomer distribution in the volume close to the substrate, as was demonstrated by Comsol simulations. Finally, the edges of the coated area contained a thin coating with vacant spots across their surface. In the case of MMA monomer deposition, the formation of droplets was only observed in the center within a small deposition area. Finally, WCA and XPS measurements provided information about the surface chemical properties of the deposits. Good agreement between surface wettability and oxygen incorporation was demonstrated. Higher hydrophilicity and higher O/C ratios were observed in zones closer to the center where better contact with the MW plasma jet was established. Based on C–O/C–C ratios calculated for all experimental conditions for both monomers it was concluded that the highest ratios were obtained at low carrier gas flow rates due to the effective fragmentation and oxidation. The obtained results with support from the literature allow for us to conclude that the styrene and MMA monomers were plasma-polymerized by the MW APPJ used. In this work, a well-established correlation between the plasma diagnostic results and coating surface properties was achieved. A high level of monomer fragmentation observed with OES, together with the non-uniform distribution of the monomer presented by CFD simulations, was shown to be a reliable indicator of coating quality. In conclusion, it can be asserted that this study gives useful insights into the properties of deposited films using an atmospheric pressure MW plasma jet with different working parameters. The carrier gas flow rate was found to have the most significant impact on the properties of the thin deposited films due to its effect on the gas flow dynamics in the system.

## Figures and Tables

**Table 1:** Summary of the selected experimental conditions. Legend: sccm, standard cubic centimeters per minute.

Sample	Power (W)	Time (s)	Distance to Substrate (mm)	Carrier Gas Flow Rate (sccm)
**Main**	10	3	5	250
**Main + t1**	10	1	5	250
**Main + t2**	10	5	5	250
**Main + p1**	7	3	5	250
**Main + p2**	13	3	5	250
**Main + f1**	10	3	5	125
**Main + f2**	10	3	5	500
**Main + f3**	10	3	5	750
**Main + d1**	10	3	3	250
**Main + d2**	10	3	7	250

**Table 2:** Emission bands/lines and their corresponding transitions, wavelengths, and selected integration time.

Band/Line	Transition	Wavelength (nm)	Integration Time (s)
OH	A-X, band 0-0	305.0–310.0	0.1
N_2_ 2nd positive	C-B, bands 0-0, 2-4, 1-3, 0-2	337.1, 370.9, 375.4, 380.4	0.1
N	^4^P_3/2_-^4^P_5/2_	824.2	1
Ar^+^	^2^D_3/2_-^2^D_3/2_	404.3	1
Ar	^2^[^3^/_2_]-^2^[^3^/_2_]°	714.7	0.1
C	^1^P°-^1^S	247.9	0.1
CH	^2^Δ-^2^Π, band 0-0	431.3	1
O	^5^P-^5^S°	777.0	0.01

**Table 3:** Thickness of the styrene-based coating under different experimental conditions.

Condition	Main	Main + t1	Main + t2	Main + p1	Main + p2	Main + f1	Main + f2	Main + f3	Main + d1	Main + d2
**Thickness (nm)**	229 ± 8	58 ± 3	486 ± 12	210 ± 5	217 ± 6	110 ± 5	220 ± 4	190 ± 10	140 ± 6	248 ± 8

**Table 4:** Surface roughness of styrene and MMA deposited thin films under different experimental conditions.

Condition/Roughness (nm)	Main	Main + t1	Main + t2	Main + p1	Main + p2	Main + f1	Main + f2	Main + f3	Main + d1	Main + d2
Styrene (zone A)	22 ± 2	23 ± 3	38 ± 5	17 ± 2	29 ± 4	22 ± 3	41 ± 2	45 ± 4	19 ± 2	20 ± 3
Styrene (zone B)	20 ± 2	14 ± 2	30 ± 4	18 ± 3	25 ± 5	15 ± 3	29 ± 6	32 ± 6	19 ± 3	20 ± 3
Styrene (zone C)	128 ± 6	63 ± 7	173 ±5	75 ± 8	149 ± 10	64 ± 4	79 ± 5	102 ± 8	125 ± 5	136 ± 8
MMA	137 ± 7	110 ± 10	156 ± 11	122 ± 10	185 ± 9	236 ± 12	110 ± 9	54 ± 5	122 ± 6	136 ± 8

**Table 5:** Water contact angle (WCA) angle values for styrene and MMA coatings prepared under different experimental conditions.

Condition	Zone A	Zone B	Zone C	MMA
Main	45.0 ± 2.0	34.2 ± 1.8	28.2 ± 3.1	26.5 ± 3.1
Main + t1	41.3 ± 3.5	31.0 ± 1.5	22.3 ± 2.3	23.7 ± 3.5
Main + t2	45.7 ± 2.1	36.5 ± 2.6	31.1 ± 2.7	30.8 ± 2.8
Main + p1	35.3 ± 3.2	20.5 ± 2.4	18.0 ± 3.3	24.4 ± 3.1
Main + p2	50.5 ± 3.5	36.3 ± 2.4	32.4 ± 3.6	35.4 ± 2.9
Main + f1	18.0 ± 1.9	12.1 ± 1.8	8.1 ± 2.1	10.8 ± 1.9
Main + f2	55.4 ± 2.5	45.8 ± 1.2	30.0 ± 2.2	37.6 ± 2.0
Main + f3	61.6 ± 2.4	52.9 ± 1.0	35.2 ± 1.7	62.0 ± 1.7
Main + d1	45.0 ± 3.6	36.1 ± 2.0	23.0 ± 3.0	28.5 ± 3.2
Main + d2	42.5 ± 2.5	33.6 ± 1.5	27.3 ± 2.3	38.0 ± 2.3

## Supplementary Materials

The following are available online at https://www.mdpi.com/2073-4360/12/2/354/s1.
