# Supplementary material for: Atmospheric Pressure Microwave Plasma Jet for Organic Thin Film Deposition
Source: Polymers (Basel). 2020 Feb 6;12(2):354. doi: 10.3390/polym12020354 (PMC7077475; doi:10.3390/polym12020354)
Supplement: Supplementary file 1 [file polymers-12-00354-s001.pdf]

Supplementary materials

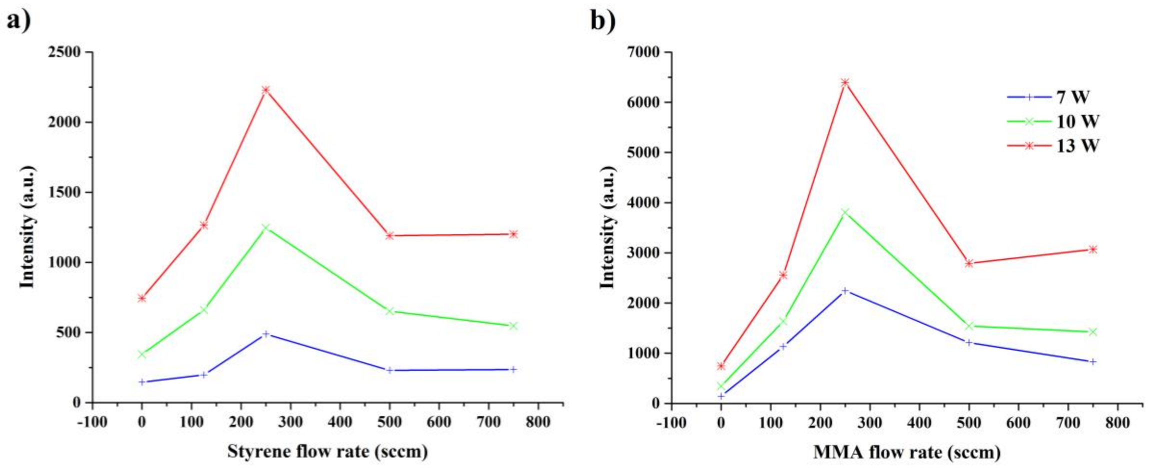

Figure S1. Dependence of the CH line emission intensity on monomer flow rate at different input powers for (a) styrene and (b) MMA monomer.

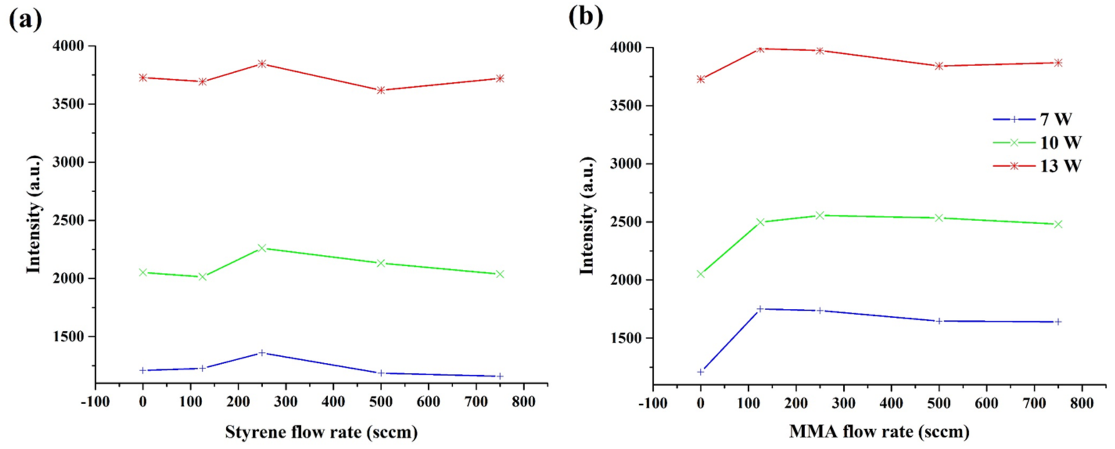

Figure S2. Dependence of the oxygen line (777 nm) emission intensity on monomer flow rate at different applied powers for (a) styrene and (b) MMA monomer.

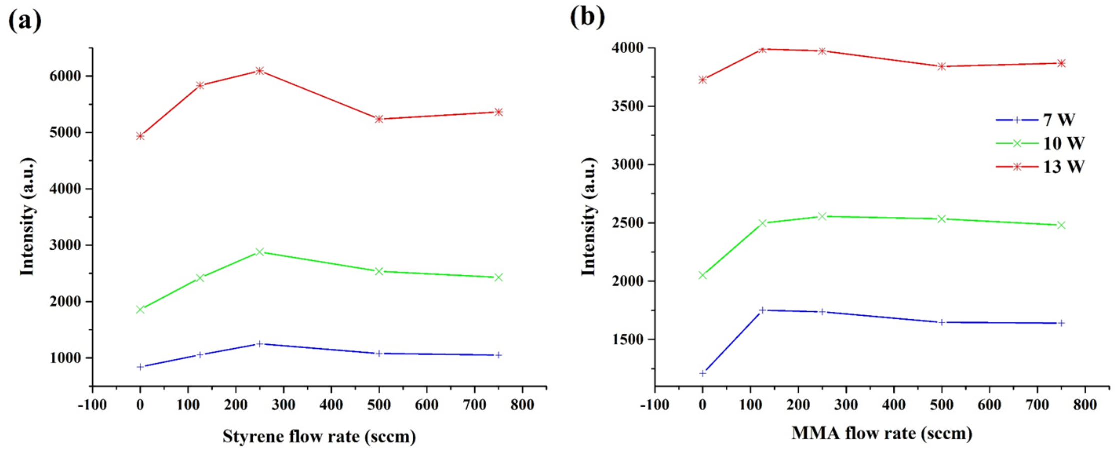

Figure S3. Dependence of the nitrogen (824.2 nm) emission intensity on monomer flow rate at different applied powers for (a) styrene and (b) MMA monomer.

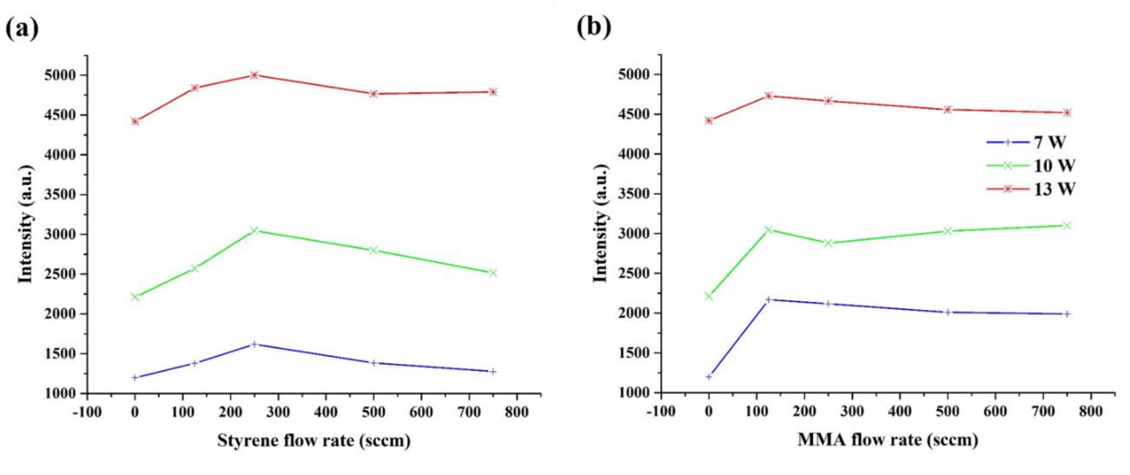

Figure S4. Dependence of the N_2 second positive system emission intensity on monomer flow rate at different powers for (a) styrene and (b) MMA monomer.

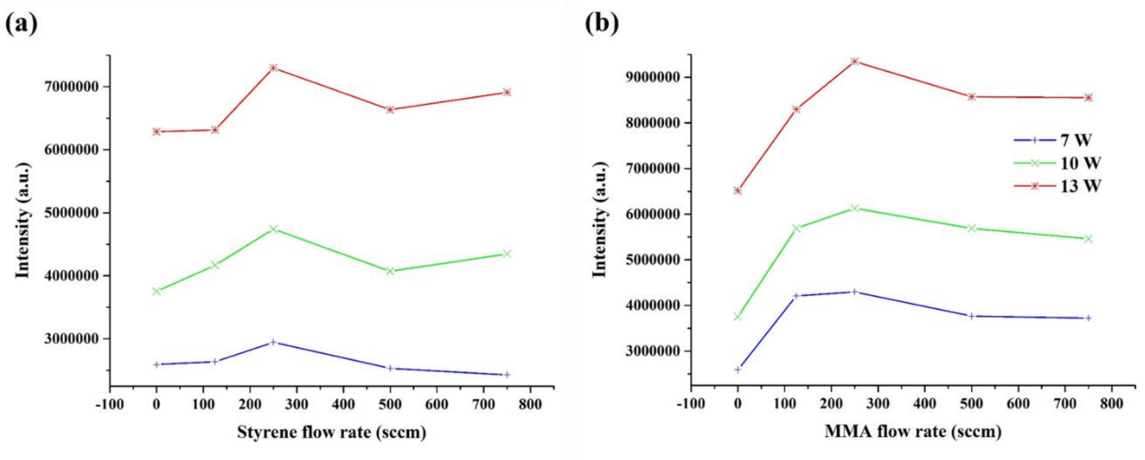

Figure S5. Dependence of the OH emission intensity on monomer flow rate at different applied powers for (a) styrene and (b) MMA monomer.

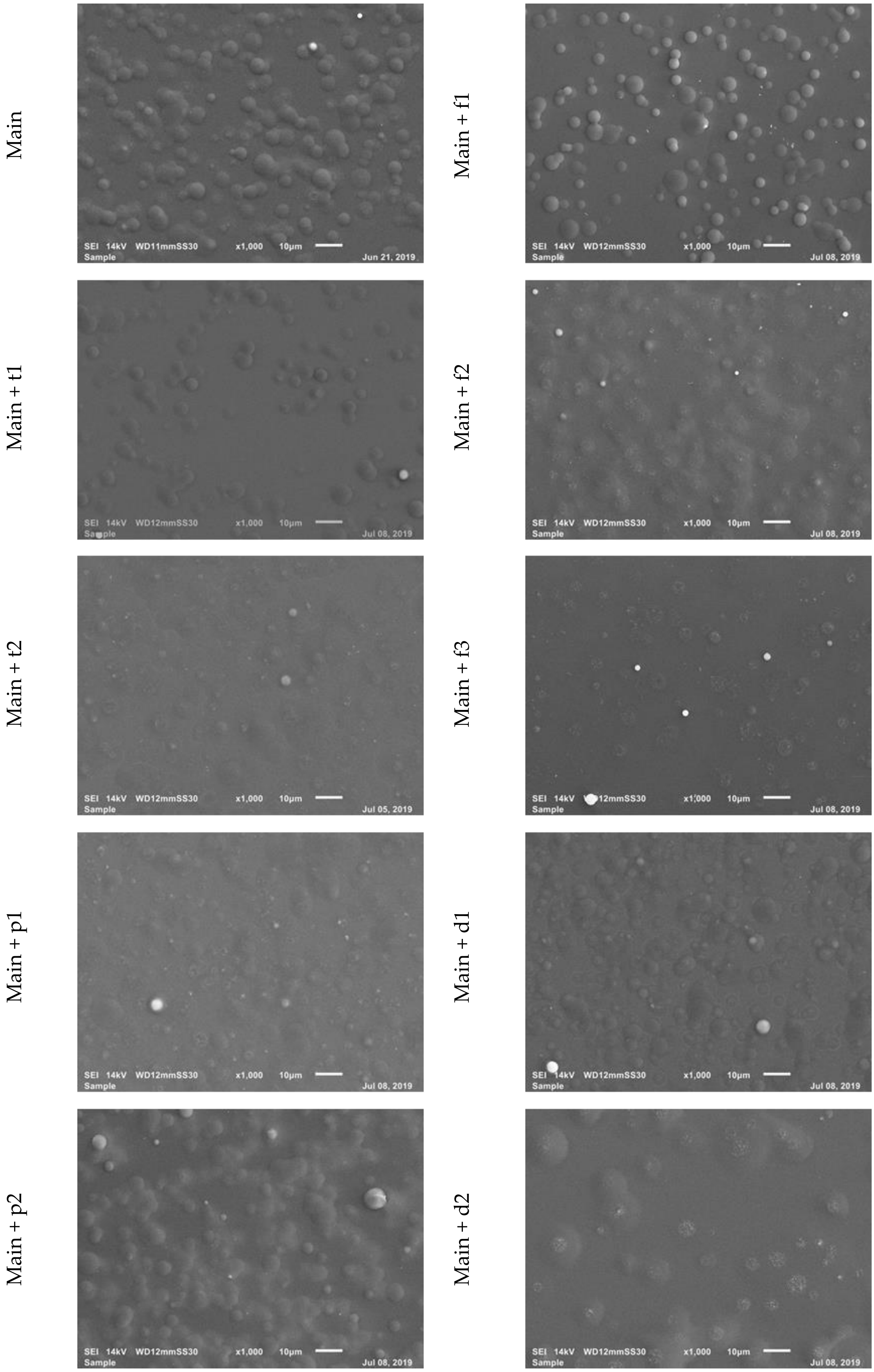

Figure S6. SEM images of the central area of the MMA coated samples under different experimental conditions.

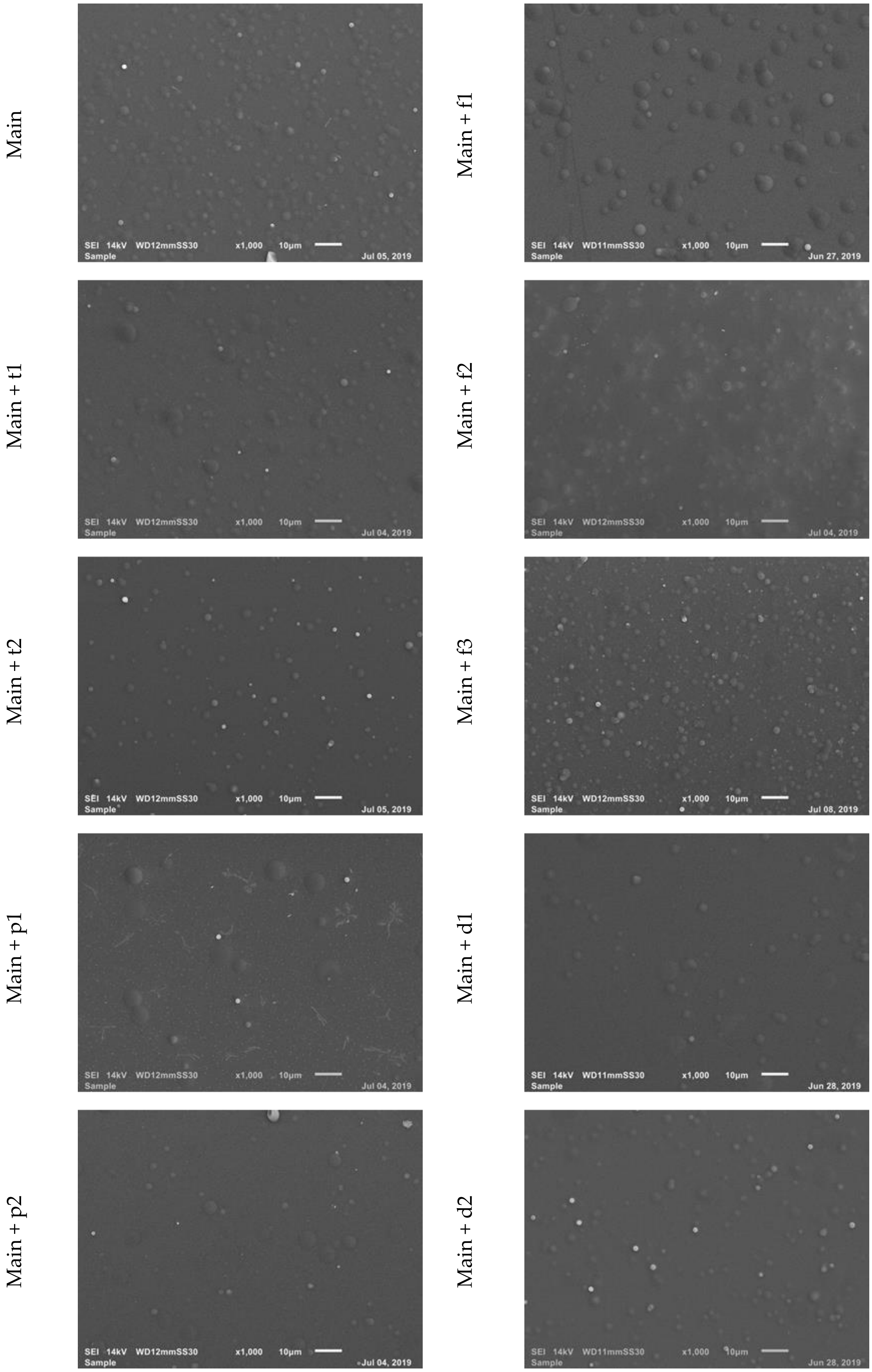

Figure S7. SEM images of the central area (zone C) of the styrene coated samples under different experimental conditions.

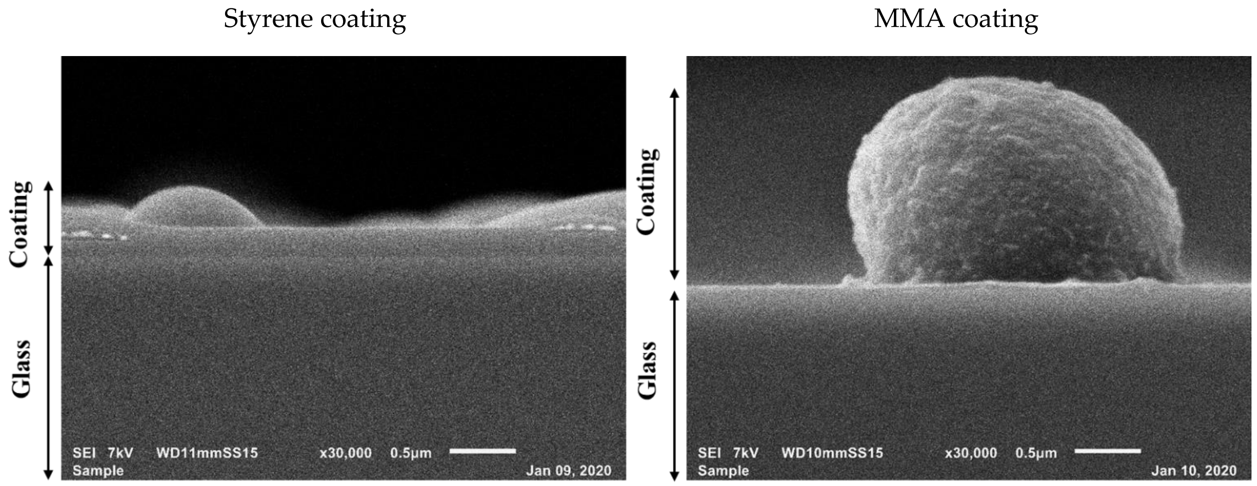

Figure S8. Cross-sectional SEM images of the styrene and MMA coated glass substrates under main condition

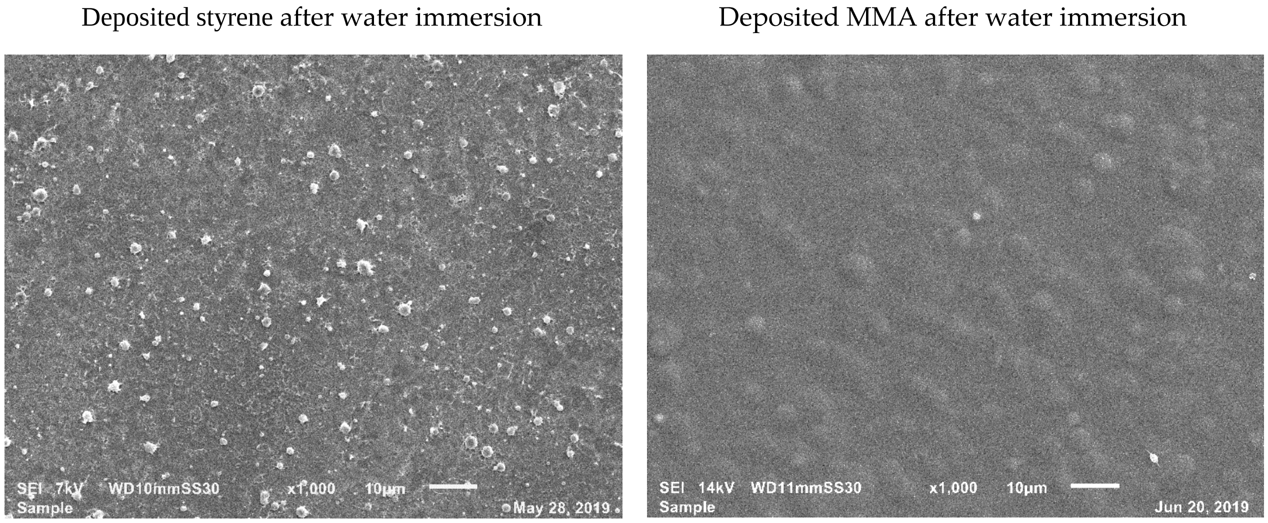

Figure S9. SEM images of the central area (zone C) of the styrene and MMA coated samples after water immersion under main condition

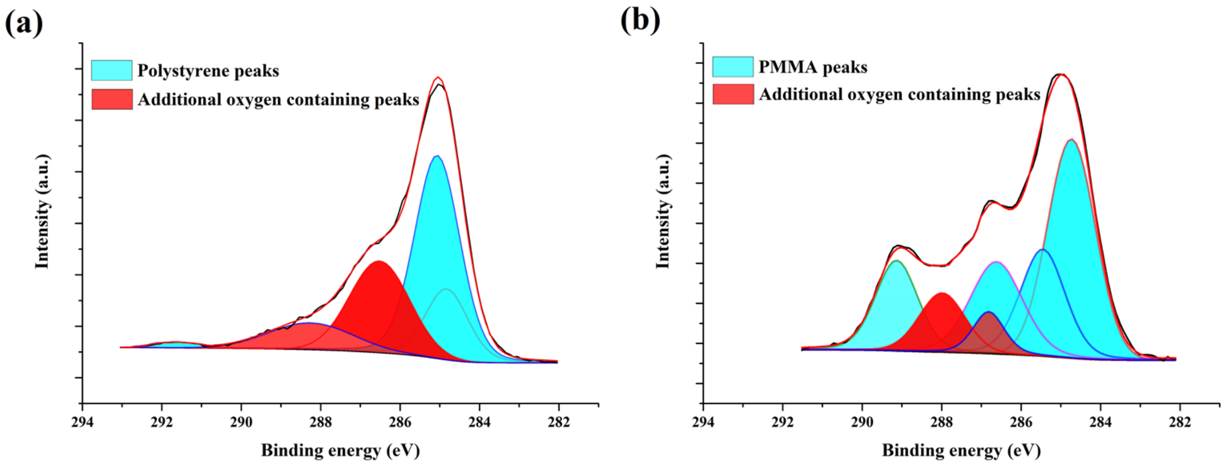

Figure S10. Deconvolution of high-resolution C1s curves for a) styrene and b) MMA deposits under main condition

Table S1. O/C ratios of the deposits calculated from the XPS elemental composition results for all experimental conditions.

		Main	Main + t1	Main + t2	Main + p1	Main + p2	Main + f1	Main + f2	Main + f3	Main + d1	Main + d2
O/C	PS (zone A)	1.16	1.15	0.88	0.5	0.5	1.23	0.24	0.2	0.24	0.22
	PS (zone B)	0.43	0.49	0.37	0.48	0.37	0.45	0.29	0.3	0.31	0.47
	PS (zone C)	0.52	0.44	0.42	0.5	0.54	1.09	0.37	0.36	0.47	0.5
	PMMA	0.81	1.15	1.17	1.19	0.24	1.9	0.7	0.6	1.47	0.62

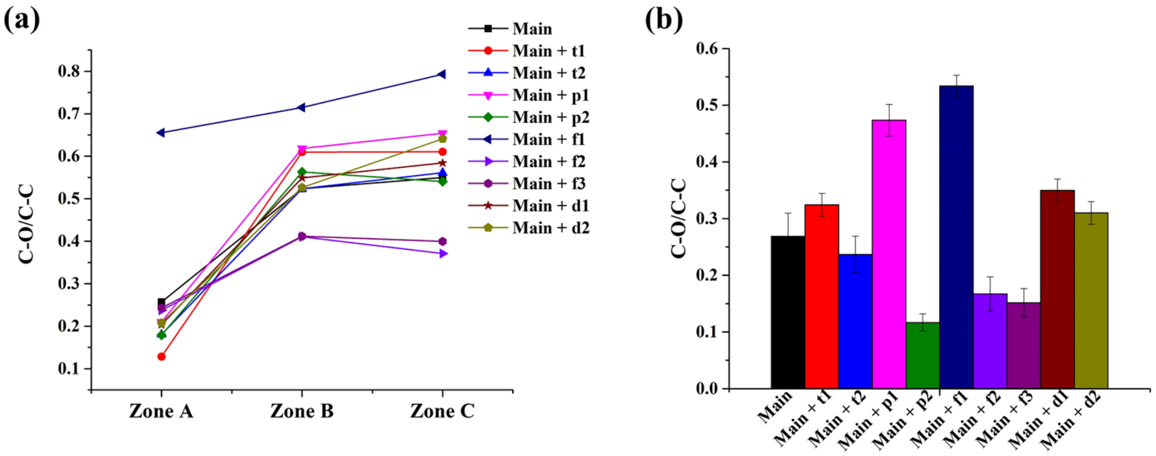

Figure S11. C-O/C-C ratio calculated for all experimental conditions for (a) styrene and (b) MMA deposited coatings.
